# Neck dissection and post-operative chemotherapy with dimethyl triazeno imidazole carboxamide and cisplatin protocol are useful for oral mucosal melanoma

**DOI:** 10.1186/1471-2407-10-623

**Published:** 2010-11-11

**Authors:** Xi Yang, Guo-xin Ren, Chen-ping Zhang, Guo-yu Zhou, Yong-jie Hu, Wen-jun Yang, Wei Guo, Jiang Li, Lai-ping Zhong

**Affiliations:** 1 Department of Oral and Maxillofacial Surgery, Ninth People's Hospital, School of Stomatology, Shanghai Jiao Tong University School of Medicine; Shanghai Key Laboratory of Stomatology, China

## Abstract

**Background:**

Oral mucosal melanoma (OMM) is a clinically rare disease with poor prognosis. Various treatment methods have been investigated with the aim of improving the prognosis. This study aimed to analyze the data of a single institution in the management of OMM.

**Methods:**

A total of 78 consecutive OMM patients were included in this retrospective study. The intraoral lesion was treated either by cryotherapy, surgery or both; the neck was treated by neck dissection or observation; post-operative chemotherapy with dimethyl triazeno imidazole carboxamide and cisplatin was performed in some patients. The Kaplan-Meier method was used for statistical analysis.

**Results:**

Among the 78 patients, there were 50 males and 28 females with an average age of 53.8 years (ranging from 27 to 85 years). The most common sites of OMM were the hard palate and gingiva. The main cause of death in OMM was distant metastasis. No significant difference was found between the intraoral/cervical lesion recurrence/post-operative distant metastasis and the intraoral lesion site/biopsy method/treatment method. The metastasis rate of cervical lymph node was high in the OMM patients, even in the patients with clinically negative necks. Cervical lesion recurrence was correlated with N stage and intraoral lesion recurrence. The survival period was longer in the patients with T3 staging, clinical stage III disease, with post-operative chemotherapy and without post-operative distant metastasis when compared to those patients with T4a staging, clinical stage IV disease, without post-operative chemotherapy and with post-operative distant metastasis.

**Conclusions:**

Radical surgery including wide intraoral resection and neck dissection is recommended for OMM patients. Post-operative chemotherapy may also be beneficial for both primary and recurrent OMM patients.

## Background

Oral mucosal melanoma (OMM) is a clinically rare malignancy arising from melanocytes within the mucosal epithelia of oral cavity. Its etiology and pathogenesis are poorly understood. OMM accounts for 0.2-8% of all melanomas and 0.26-0.5% of oral malignancies [1-4], and occurs with a higher incidence in adults aged sixties, without any gender preference. The most common sites of OMM are the hard palate and maxillary gingiva. The prognosis of OMM is poor with a very low 5-year survival rate (about 10-25% or even lower) [1-4]. Clinically, OMM may present as a macular or nodular lesion with its surface color ranging from brown, grey, black and white to purple and red shade or even depigmented [2,3,5]. Satellite lesions frequently surround the initial tumor. Cervical lymph node metastasis (CLNM) and distant metastasis often occur in OMM patients with distant metastasis being the main cause of death [6]. As such, early diagnosis and treatment is the key in improving the prognosis. Surgery has been the mainstay treatment for OMM, and radical resection with a wide margin is always recommended. Other treatment modalities have included chemotherapy [dimethyl triazeno imidazole carboxamide (DTIC), platinum analogs, vincristine, and nimustine hydrochloride], radiotherapy, vaccine-based therapy, and immunotherapy (interleukin-2 and interferon) [7-11]. Because of the poor prognosis in advanced disease, palliative care may be the only option in such cases. Although much effort has been directed towards modifying treatment protocols so as to improve the prognosis of OMM patients, some treatment issues are still under debate. These include the efficacy of cryotherapy in controlling the intraoral lesion, the need for neck dissection and the decision of which post-operative chemotherapy protocol to implement. Recently, mucosal melanoma of the head and neck has been added to the new AJCC-TMN classification (Additional file 1) [12]. In contrast to the previous TMN classification, under this new classification, tumor size T1 and T2 and clinical stage I and II are omitted because of the aggressiveness of this disease. Regarding tumor size, for T3 lesions, they are located in the epithelium or submucosa, just as in mucosal disease. For T4a lesions, they are located in the deep soft tissue, cartilage, bone, or overlying skin. For T4b lesions, they have invaded the brain, dura, skull base, lower cranial nerves, masticator space, carotid artery, prevertebral space, mediastinal structures, cartilage, skeletal muscle, or bone. Regarding stage grouping, stage III is T3N0M0, stage IVA is T4aN0M0 and T3-4aN1M0, stage IVB is T4bAnyN and stage IVC is AnyTAnyNM1. In the current study, we presented 78 consecutive OMM cases (64 primary OMMs and 14 recurrent OMMs) and have also discussed the various aspects of intraoral lesion management, neck management and post-operative chemotherapy with the protocol of DTIC and cisplatin (also known as cis-diaminedichloroplatinum, CDDP).

## Methods

From January 2004 to January 2010, 78 consecutive OMM patients, including 64 primary OMMs and 14 recurrent OMMs, were retrospectively reviewed with their clinical data (Additional file 2). These patients were treated at the Department of Oral and Maxillofacial Surgery, Ninth People's Hospital, School of Medicine, Shanghai Jiao Tong University. From the clinical data, routine clinical examination was performed in all patients. In addition, computerized tomography (CT) of the craniomaxillofacial region, neck, and lung was also performed. ^99^Tc emission computerized tomography of entire body was performed in patients in whom bony distant metastasis was suspected. PET-CT was initially recommended in such cases, but the majority of these patients refused this due to economic reasons. After signing the informed consent forms, all patients with primary or recurrent OMMs received pre-operative incisional or excisional biopsy of the intraoral lesion at room temperature or pre-operative incisional biopsy during cryotherapy or intra-operative frozen section for pathological diagnosis.

For intraoral lesions, the pre-operative incisional or excisional biopsy was performed under local anesthesia with 1% lidocaine (Lidocaine Hydrochloride Injection, Shanghai Fuda Pharmaceutical Ltd, Shanghai, China). Routine pathological examination was performed using hematoxylin and eosin (HE) staining; if the diagnosis was unclear, immunohistochemical examination was performed using the S-100, Vimentin, HMB45 and MelanA antibodies, which would show positivity for melanoma. The incisional biopsy during cryotherapy or intra-operative frozen section was performed under local anesthesia with 1% lidocaine; frozen pathological examination was performed using HE staining. If the diagnosis was unclear, further specimens were taken until a definite pathological diagnosis was obtained. As part of the neck examination, if CT scan showed CLNM, fine needle aspiration cytology (FNAC) examination was not suggested; if in clinically suspicious CLNM cases whereby CLNM could not be verified by CT scan, FNAC examination was performed on the suspicious lymph node.

For patients with clinical stage III disease, cryotherapy was initially suggested for the treatment of intraoral lesions. However, when the lesion was large or in cases whereby bony exposure occurred after cryotherapy, surgical resection was performed. Surgery was also indicated if patients had refused cryotherapy. For patients with clinical stage IVA disease, cryotherapy and surgical resection were both performed. In such cases, the mucosal or submucosal lesion was treated by cryotherapy while the invaded deep structures were removed by surgery. Similarly, surgery was performed if patients refused cryotherapy. For patients with clinical stage IVB and IVC disease, chemotherapy with/without radiotherapy was recommended. Post-operative chemotherapy was recommended in patients with positive surgical margins.

Cryotherapy was performed under local anesthesia with 1% lidocaine. A special cryospray unit (HX17-YDQ-500, Beijing Western Vision Technology Co., Ltd., Beijing, China) was used to spray liquid nitrogen on to the oral mucosa to destroy the involved lesion of oral mucosa as well as 0.5 cm marginal mucosa. If the intraoral lesion could not be confidently controlled, surgical operation was performed. In cases whereby safety margins could not be achieved due to functional and aesthetic limitations, intra-operative cryotherapy was performed at these resection margins.

Surgical resection was performed in the majority of OMMs patients. For intraoral lesions, an extended resection with a 2 cm safety margin was performed. For lesions of the hard palate and maxillary gingiva, extended resection with subtotal maxillectomy for T3 lesions and total maxillectomy with or without preservation of the orbital floor for T4a lesions was preformed. For lesions of the mandibular gingiva, extended resection with marginal mandibulectomy for T3 lesions and segmental mandibulectomy for T4a lesions was performed. Radical neck dissection was planned in patients with clinically positive CLNM while functional neck dissection was planned in patients with clinically negative CLNM.

The following chemotherapy protocol of DTIC (Dacarbazine Injection, Nanjing Pharmaceutical Factory Co. Ltd., Jiangsu, China) and CDDP (cisplatin Injection, Qilu Pharmaceutical Co. Ltd., Shandong, China) was used: 80 mg/m^2 ^of CDDP on the first day, 250 mg/m^2 ^of DTIC from the first day to the fifth day. After two weeks, patients received a second cycle of chemotherapy (21 days of each cycle). Two to four cycles were recommended; if response was stable disease, more cycles were used; if patients could not tolerate the complications of chemotherapy, chemotherapy was stopped.

For patients with recurrent OMMs, extended resection with post-operative chemotherapy was performed. The chemotherapy protocol was the same as previously described. Post-operative radiotherapy was recommended for patients whose disease could not be controlled by surgery and post-operative chemotherapy.

Follow-up reviews were carried out for all patients after the initial treatment: every 2-4 months during the first year, every 4-6 moths during the second year, and then twice a year. Besides physical examinations, CT scans of the craniomaxillofacial region, neck, and lung and ^99^Tc emission computerized tomography of entire body were performed every half a year. PET-CT was performed if there was suspicion of distant metastasis to lung, brain or bones. If there was a suspicion of a recurrent intraoral lesion, incisional or excisional biopsy was performed. All data was analyzed using SPSS 13.0 for Windows (SPSS Inc., USA), and the survival analysis was performed using the Kaplan-Meier method. Statistically significance was set at P < 0.05.

## Results

The clinical records of 78 patients were analyzed and were presented in Table 1. The sample consisted of 50 males and 28 females with an average age of 53.8 years (ranging from 27 to 85 years). With regards to the site of lesion, 35 lesions originated from the hard palatal mucosa, 20 lesions from the maxillary gingival mucosa, 16 lesions from the mandibular gingival mucosa, three lesions from the buccal mucosa, three lesions from the lip mucosa and one lesion from the mucosa of the floor of mouth. From the sample of 78 patients, 64 patients presented with primary OMMs while 14 patients presented with recurrent OMMs. The disease course of primary OMM patients ranged from 1 to 48 months with a mean of 6.6 months, while that of recurrent OMM patients ranged from 1 to 36 months with a mean of 2.8 months. The majority of patients presented with local swelling and surface pigmentation (black, brown, grey, purple or red shades) with only three patients presenting with local swelling without surface pigmentation. Other surface characteristics of OMM included a smooth surface or ulceration with bleeding and pain and occasional tooth loosening. 35 patients (26 patients with primary OMMs and 9 patients with recurrent OMMs) presented with enlarged cervical lymph nodes.

**Table 1:** Clinical parameters of 78 patients with oral mucosal melanoma

Clinical parameters	Number of patients (%)
Primary patients	64
Sex	
Male	41 (64.1)
Female	23 (35.9)
Age (years old)	
< 30	1 (1.6)
30-39	10 (15.6)
40-49	16 (25)
50-59	27 (42.2)
60-69	11 (17.2)
70-79	11 (17.2)
≥ 80	2 (3.1)
Intraoral site	
Hard palate	28 (43.8)
Maxillary gingiva	15 (23.4)
Mandibular gingiva	14 (21.9)
Buccal mucosa	3 (4.7)
Lip	3 (4.7)
Floor of mouth	1 (1.6)
T stage	
T3	53 (82.8)
T4a	11 (17.2)
N stage	
cN0	38 (59.4)
cN1	26 (40.6)
pN-	25 (39)
pN0	3 (4.7)
pN1	36 (56.3)
M stage	
M0	62 (96.9)
M1	2 (3.1)
Clinical stage	
III	22 (34.4)
IVA	40 (62.5)
IVC	2 (3.1)
Recurrent patients	14
Sex	
Male	9 (64.3)
Female	5 (35.7)
Type of recurrence	
Intraoral recurrence	5 (35.7)
Intraoral and neck recurrence	9 (64.3)

### Primary OMM

According to the AJCC-TNM classification for the mucosal melanoma of the head and neck [12], among the 64 patients with primary OMMs in our study, pertaining to T staging, 53 patients presented with stage T3 while 11 patients presented with stage T4a. With regard to clinical N staging, 38 patients presented with stage cN0 with 26 patients presenting with stage cN1. For distant metastasis M staging, 62 patients presented with stage M0 with the remaining two patients presenting with stage M1. Among the 39 patients who received neck dissection, three patients were staged pN0 while 36 patients were staged pN1. As such, with regards to stage grouping, 22 patients were at clinical stage III, 40 patients were at clinical stage IVA and two patients were at clinical stage IVC. During the follow-up period from 3 to 67 months (with a mean of 35.5 months), 12 patients were lost. The overall survival rate of the patients with primary OMMs was 61.5%, and the 1-, 2-, 3- and 5-year survival rate was 58.3%(28/48), 53.1%(17/32), 35.0%(7/20) and 36.4%(4/11), respectively. The local lesion recurrence rate was 30.7%, while the recurrence rate of late CLNM was 15.4%. Our data showed that there was no significant difference in survival rate between the different distributions of sex, age, course of disease and primary lesion site.

#### Local lesion management

Three types of intraoral lesion treatment were performed and this included cryotherapy only (36 cases), surgery only (11 cases), cryotherapy and surgery (17 cases), respectively. Among the 52 patients with complete follow up data, no significant relationship between the intraoral lesion recurrence and the lesion site (P = 0.39), T stage (P = 0.49), biopsy method (P = 0.18), intraoral lesion treatment method (P = 0.50), or post-operative chemotherapy (P = 0.23) was found. However, the intraoral lesion recurrence rate was somewhat higher in the patients with cryotherapy than that in the patients without cryotherapy (Table 2). The mean survival period of patients with T3 staging was 48.4 ± 4.2 (95% CI: 40.2-56.6) months, which was significantly longer (Log Rank = 20.315, P < 0.001) than that of patients with T4a staging [11.3 ± 1.4 (95% CI: 8.6-14.0) months]. There was no significant difference in survival rates among patients with different intraoral lesion treatment methods (Log Rank = 1.561, df = 2, P = 0.46). We found no significant difference in survival rates between patients with and without intraoral lesion recurrence (Log Rank = 0.020, P = 0.89).

**Table 2:** Th treatment results between the primary intraoral lesion stage and treatment methods

Classification	Treatment methods	Case number	Late intraoral recurrence	Late cervical lymph node metastasis	Late distant metastasis
T3 stage	Cryotherapy	33	6/26 (23.1%)	5/26 (19.2%)	11/26 (42.3%)
	Surgery	7	1/5 (20%)	0/5	0/5
	Both	13	5/11 (45.5%)	1/11 (9.1%)	1/11 (9.1%)
T4a stage	Cryotherapy	3	2/3 (66.7%)	2/3 (66.7%)	2/3 (66.7%)
	Surgery	4	1/4 (25%)	0/4	3/4 (75%)
	Both	4	1/3 (33.3%)	0/3	2/3 (66.7%)

#### Neck management

From the 64 patients with primary OMM, twenty-five patients refused a neck dissection, including 22 patients with clinically negative CLNM and three patients with clinically suspicious positive CLNM. During the follow-up period, five patients were lost, and late CLNM occurred in three patients of the remaining 20 patients (15%, 3/20).

The remaining thirty-nine patients with primary OMM received a neck dissection, including 23 patients with clinically positive CLNM and 16 patients with clinically negative CLNM. Among the 16 patients with clinically negative CLNM, pathological CLNM was confirmed in 13 patients (81.3%, 13/16). Our data showed that the rate of CLNM in the group with neck dissection was significantly higher than that in the group without neck dissection (χ^2 ^= 15.8, P < 0.001). Among the 23 patients with clinically positive CLNM, pathological CLNM was confirmed in all patients (100%, 23/23), with six patients showing extra-capsular spread of lymph nodes. During the follow-up period, seven patients were lost, with late CLNM occurring in five of the remaining 32 patients (15.6%, 5/32). The incidence of late CLNM was significantly correlated with N staging (Z = -2.2, P = 0.027) and the incidence of recurrence of the intraoral lesion (Z = -2.1, P = 0.036). Furthermore, we found that the incidence of late CLNM in patients with positive CLNM and recurrence of the intraoral lesion was significantly higher than those patients with negative CLNM and without late recurrence of the intraoral lesion. In clinically CLNM-negative patients, the incidence of late CLNM in patients confirmed with pathological CLNM was significantly higher than those without pathological CLNM (Z = -2.7, P = 0.007). No significant relationship between late CLNM and the lesion site (P = 0.79), T stage (P = 0.66), biopsy method (P = 0.65), intraoral lesion treatment method (P = 0.93), neck dissection method (P = 0.34), extra-capsular spread (P = 0.25), post-operative distant metastasis (P = 0.10) or post-operative chemotherapy (P = 0.44) was found.

The mean survival period of patients with pathologically CLNM was 37.2 ± 5.3 (95% CI: 26.9-47.5) months and this was shorter than that of those without CLNM [49.0 ± 6.0 (95% CI: 37.3-60.7) months], although this difference was not significant (Log Rank = 2.609, P = 0.106). However, if the status of CLNM was based on clinical examination, the difference in survival period between patients with and without CLNM was significant (Log Rank = 8.08, P = 0.004), and the survival period of patients with CLNM [29.8 ± 6.6 (95% CI: 16.9-42.7) months) was shorter than that of those without CLNM [50.0 ± 4.9 (95% CI: 40.5-59.6) months]. The mean survival period of patients with extra-capsular spread of lymph nodes was 21.2 ± 5.1 (95% CI: 11.1-31.2) months, which was shorter than that of those without extra-capsular spread of lymph nodes [39.8 ± 5.7 (95% CI: 28.7-51.0) months]. However, this difference was not significant (Log Rank = 0.383, P = 0.54). Late CLNM occurred in eight patients, but the difference in survival rate was not significant between patients with and without late CLNM (Log Rank = 0.032, P = 0.86). In addition, the difference in survival rate was not significant between patients with and without neck dissection (Log Rank = 1.919, P = 0.166).

#### Distant metastasis

Two patients presenting with distant metastasis before treatment died at the 6th and 15th month after surgical treatment. The survival period of the patients without distant metastasis was 43.7 ± 4.1 (95% CI: 35.6-51.7) months, and the difference between these two groups of patients was significant (Log Rank = 7.341, P = 0.007).

**Figure 1:**
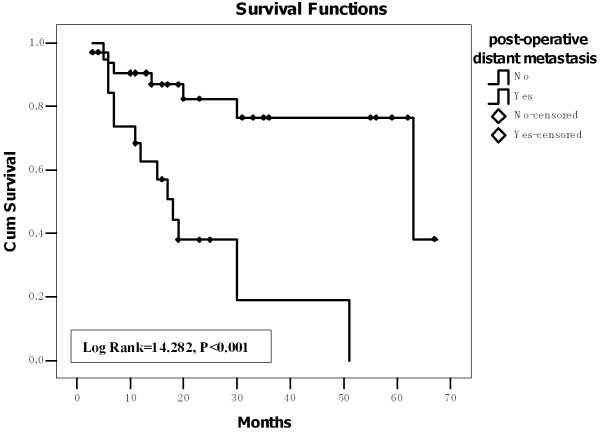
**The mean survival period of the patients with post-operative distant metastasis was significant shorter than that of those without post-operative distant metastasis (Kaplan-Meier analysis)**.

Post-operative distant metastasis occurred in 17 patients. The site of distant metastasis was lung only (10 patients), brain only (two patients) and multi-sites including lung, brain, breast, liver and bones (five patients). Figure 1 shows that the mean survival period of the patients with post-operative distant metastasis was 22.6 ± 4.7 (95% CI: 13.4-31.7) months, which was significant shorter (Log Rank = 14.282, P < 0.001) than that of those without post-operative distant metastasis [53.4 ± 4.4 (95% CI: 44.8-61.9) months].

#### Clinical stage

**Figure 2:**
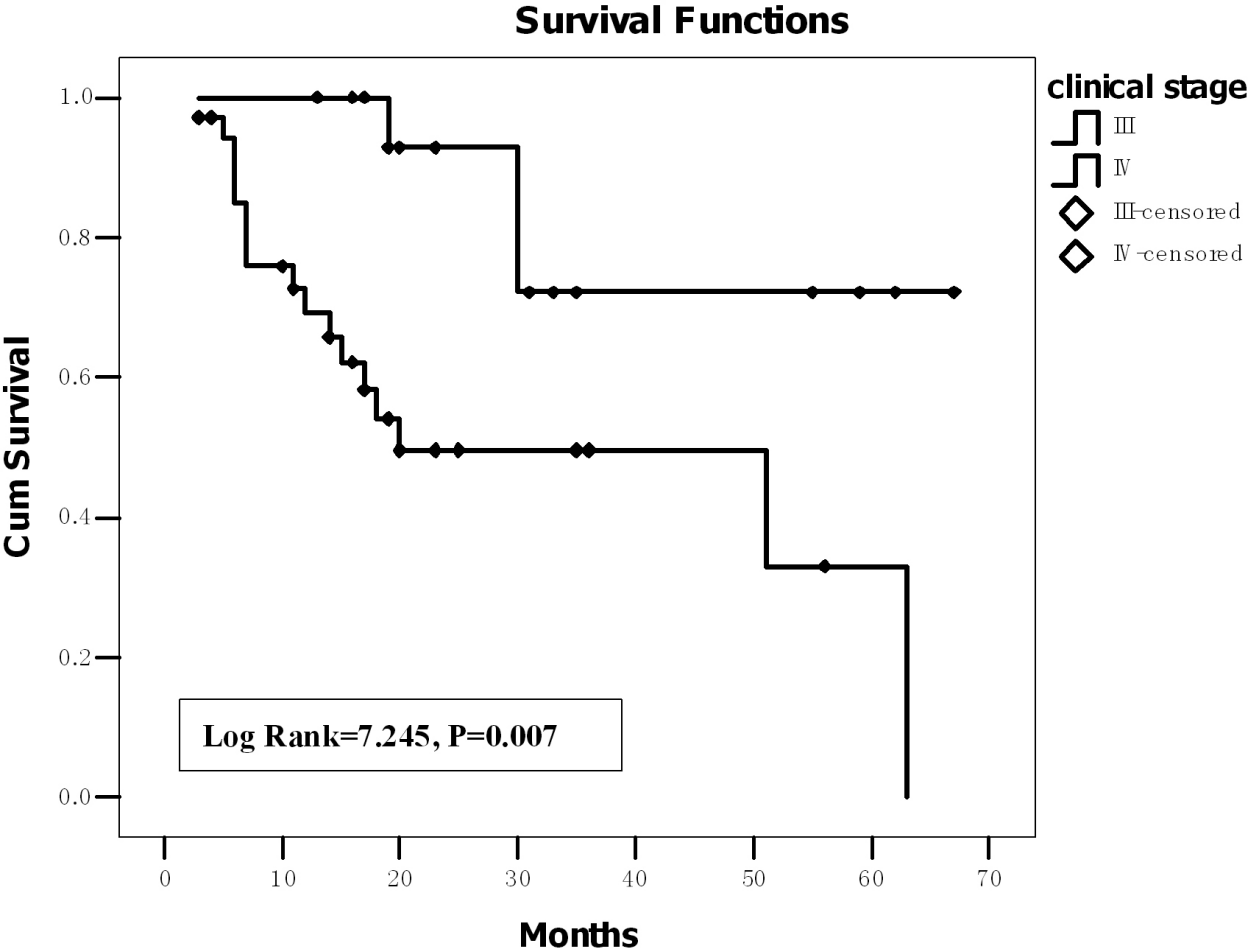
**The mean survival period of the patients at the clinical stage III was significantly longer than that of those at the clinical stage IV (Kaplan-Meier analysis)**.

Figure 2 shows that the mean survival period of patients with clinical stage III disease was 55.9 ± 5.5 (95% CI: 45.2-66.7) months, which was significantly longer than that of those with clinical stage IV disease [34.8 ± 5.0 (95% CI: 25.1-44.5) months] (Log Rank = 7.245, P = 0.007).

#### Post-operative chemotherapy

**Figure 3:**
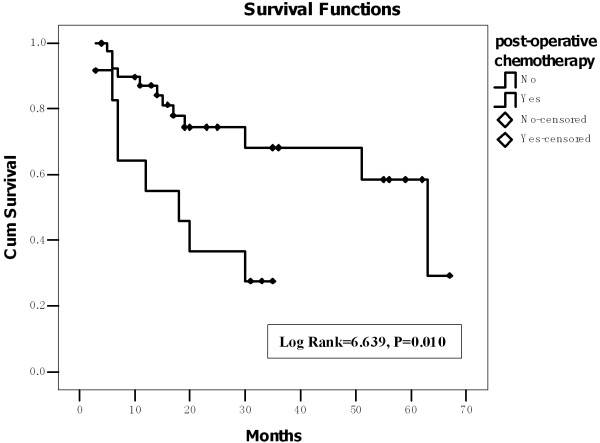
**The mean survival period of the patients with post-operative chemotherapy was significantly longer than that of those without post-operative chemotherapy(Kaplan-Meier analysis)**.

Forty patients received post-operative chemotherapy. Figure 3 shows that the mean survival period of patients with post-operative chemotherapy was 47.8 ± 4.4 (95% CI: 39.2-56.4) months, which was significantly longer (Log Rank = 6.639, P = 0.010) than that of those without post-operative chemotherapy [19.0 ± 3.7 (95% CI: 11.8-26.2) months]. The survival rate of patients with two to four cycles of chemotherapy was 76.7% (23/30). While the survival rate of patients with one or more than four cycles of chemotherapy was both 40%, the survival rate of those without chemotherapy was 33.3% (4/12). Among the patients without neck dissection, the mean survival period of patients with post-operative chemotherapy was 53.8 ± 6.6 (95% CI: 40.8-66.9) months, which was longer than that of those without post-operative chemotherapy [21.8 ± 5.3 (95% CI: 11.3-32.2) months], although this difference was not significant (Log Rank = 3.249, P = 0.071). However, the post-operative chemotherapy could benefit the patients (both with and without post-operative distant metastasis) on the survival period (Log Rank = 4.927, P = 0.026) compared to the patients without post-operative chemotherapy.

#### Post-operative radiotherapy

Post-operative radiotherapy was used in two patients in whom the intraoral and neck lesions could not be controlled by surgery and post-operative chemotherapy. These two patients died within the first year of follow-up period.

#### Treatment protocol

Our study summarized four types of treatment protocols. This consisted of intraoral lesion treatment only (cryotherapy) (five cases), intraoral lesion treatment (cryotherapy, surgery or both) with neck dissection (14 cases), intraoral lesion treatment (cryotherapy or cryotherapy with surgery) with chemotherapy (20 cases), and intraoral treatment (cryotherapy, surgery or both) with neck dissection and chemotherapy (25 cases). The data showed a significant difference of survival rate among these four treatment protocols (Log Rank = 8.445, df = 3, P = 0.038).

### Recurrent OMM

Among the 14 patients with recurrent OMM, five patients were diagnosed with intraoral lesion recurrence (three hard palatal mucosa and two maxillary gingival mucosa), and nine patients were diagnosed with intraoral lesion recurrence and CLNM (four hard palatal mucosa, three maxillary gingival mucosa and two mandibular gingival mucosa). Cryotherapy only, surgery only and cryotherapy with surgery were performed in six, five and three patients, respectively. A total of 13 patients received a neck dissection while the remaining patient with a recurrent intraoral lesion refused a neck dissection. Pathological CLNM was confirmed in 12 patients (including all of the nine patients with intraoral lesion recurrence and CLNM). No significant relationship was found among the clinico-pathological parameters. During the follow-up period, one patient was lost while the remaining 13 patients were followed-up from 10 to 67 months with a mean of 35.4 months. In these patients, the overall survival rate of patients with recurrent OMM was 38.5%, the intraoral lesion recurrent rate was 15.4%, and the incidence of late CLNM was 46.2%.

## Discussion

At present, the cause of OMM remains unclear, and the prognosis is still poor. The management of OMM remains a big challenge. In our study, the survival period of patients with clinical stage III disease was significantly longer than those with clinical stage IV disease. For the patients with primary OMM, the overall survival rate was 61.5%, and the 1-, 2-, 3- and 5-year survival rates were 58.3%, 53.1%, 35.0%, and 36.4%, respectively. From our results, regardless of whether cryotherapy was used, we recommend that radical surgery including wide intraoral resection and neck dissection to be the first choice of treatment.

Although chemotherapy is commonly used in the treatment in malignancies, the therapeutic value of chemotherapy on melanoma has not been previously reported [13]. Recently, chemotherapy has been used for OMM patients, and many different types of chemotherapeutic agents have been reported, such as DTIC, CDDP, Vincristine, Nimustine, Temozolomide, Tamoxifen [14]. Combining chemotherapeutic agents such as DTIC and CDDP has also been reported [15-17]. In this study, a post-operative chemotherapy protocol of DTIC with CDDP was implemented for patients with both primary and recurrent OMMs. The survival period of patients with post-operative chemotherapy was significantly longer than that of those without post-operative chemotherapy. As such, the implementation of a post-operative chemotherapy protocol with DTIC and CDDP could be beneficial in terms of survival for OMM patients. In addition, two to four cycles of post-operative chemotherapy was found to improve survival rates when compared to other cycle regimes. However, further prospective studies are needed to confirm the efficacy of post-operative chemotherapy in the treatment of OMM.

Neck dissection is strongly recommended in OMM patients with clinically positive CLNM [18], because CLNM is significantly correlated with survival rate in the OMM patients [19,20]. However, there is still some controversy on the issue of neck dissection in the clinically CLNM-negative patients. In our study, 16 clinically CLNM-negative patients received neck dissection, and 81.3% of these patients were confirmed with pathological CLNM. In contrast, among the 20 clinically CLNM-negative patients who did not receive a neck dissection, pathological CLNM was confirmed in three patients (15%). In these 36 clinically CLNM-negative patients, the incidence of pathological CLNM was at least 44.4%. As such, because of this high incidence, neck dissection is recommended in OMM patients. As far as the type of neck dissection method to be performed, functional neck dissection should be performed in the clinically CLNM-negative patients while radical neck dissection should be performed in the clinically CLNM-positive patients.

The use of cryotherapy has been reported in the treatment of cutaneous melanoma [21], but seldom reported in the treatment of OMM [22]. In our study, cryotherapy was used to treat intraoral lesions. Although the intraoral lesion recurrence rate was not significant between the various treatment methods of cryotherapy, surgery or both, the intraoral lesion recurrence rate in patients with cryotherapy was slightly higher than those who received surgery. Based on our experience, either cryotherapy or surgery alone is beneficial, in terms of the intraoral lesion recurrence rate, in patients with T3 lesions or smaller-sized lesions. Even though cryotherapy has been used as the primary treatment, inadequate margin control or recurrence of the intraoral lesion may occur. As such, surgical resection should always be considered in these cases to completely remove the intraoral lesion. For patients with T4a lesions or even larger-sized T3 lesions, cryotherapy alone is not sufficient to control the disease. Instead, surgical resection is strongly recommended.

There is still some controversy on the use of post-operative radiotherapy in OMM patients. Previous studies have reported that the local control rate after surgery and radiotherapy is higher than either surgery or radiotherapy alone, although the difference in survival was not significant [23,24]. Furthermore, mucosal melanoma is usually regarded as a radiation-resistant tumor [25,26]. On the other hand, some authors have reported on the benefits of radiotherapy alone in the treatment of mucosal melanoma [20,22,27-29]. In our study, post-operative radiotherapy was not routinely used for OMM patients. Although post-operative chemotherapy was implemented in the majority of patients, the efficacy of concurrent post-operative chemotherapy and radiotherapy in improving the prognosis of OMM should be further investigated in prospective trials.

## Conclusions

OMM is a rare disease with a clinically poor prognosis. Radical surgery including wide intraoral resection and neck dissection is recommended as the primary treatment modality. In addition, a post-operative chemotherapy protocol of DTIC and CDDP may be beneficial in the treatment both primary and recurrent OMMs.

## Competing interests

The authors declare that they have no competing interests.

## Authors' contributions

LZ and CZ were responsible for the study design, interpretation of the data and revision of the manuscript. XY and GR were responsible for data acquisition, analysis of the work presented and the preparation of the manuscript. GZ, YH, WY, WG and JL participated in the clinical management of the patients. All authors read and approved the final manuscript.

## Pre-publication history

The pre-publication history for this paper can be accessed here:

http://www.biomedcentral.com/1471-2407/10/623/prepub

## Supplementary Material

Additional file 1 **AJCC-TNM classification for the mucosal melanoma of the head and neck**. This table contains detail information of TNM classification for the mucosal melanoma of the head and neck (in AJCC cancer staging manual, 7th Edition, published in 2010). Click here for file

Additional file 2 **Clinical data of 78 OMM patients**. This table contains detail information of each OMM patient including individual information, symptoms, TNM staging, biopsy method, treatment protocol, and prognosis. Click here for file
