# Supplementary material for: Neck dissection and post-operative chemotherapy with dimethyl triazeno imidazole carboxamide and cisplatin protocol are useful for oral mucosal melanoma
Source: BMC Cancer. 2010 Nov 11;10:623. doi: 10.1186/1471-2407-10-623 (PMC2993680; doi:10.1186/1471-2407-10-623)
Supplement: Additional file 1 — AJCC-TNM classification for the mucosal melanoma of the head and neck. This table contains detail information of TNM classification for the mucosal melanoma of the head and neck (in AJCC cancer staging manual, 7th Edition, published in 2010). [file 1471-2407-10-623-S1.DOC]

**Supplemental table 1:** AJCC-TNM classification for the mucosal melanoma of the head and neck

| **Primary Tumor** | | | |
| --- | --- | --- | --- |
| T3 | Mucosal disease | | |
| T4a | Moderately advanced disease. Tumor involving deep soft tissue, cartilage, one, or overlying skin | | |
| T4b | Very advanced disease. Tumor involving brain, dura, skull base, lower cranial nerves (IX, X, XI, XII), masticator space, carotid artery, prevertebral space, or mediastinal structures | | |
| **Regional Lymph Nodes** | | | |
| NX | Regional lymph nodes cannot be assessed | | |
| N0 | No regional lymph node metastases | | |
| N1 | Regional lymph node metastases present | | |
| **Distant Metastasis** | | | |
| M0 | No distant metastasis | | |
| M1 | Distant metastasis present | | |
| **Clinical stage** | | | |
| Stage III | T3 | N0 | M0 |
| Stage IVA | T4a | N0 | M0 |
|  | T3-T4a | N1 | M0 |
| Stage IVB | T4b | Any N | M0 |
| Stage IVC | Any T | Any N | M1 |
